# C-reactive Protein Trends and Clinical Outcomes in Patients Treated for Complicated Parapneumonic Effusion and Empyema

**DOI:** 10.7759/cureus.98802

**Published:** 2025-12-09

**Authors:** John Ferguson

**Affiliations:** 1 Critical Care Medicine, Critical Care and Pulmonary Consultants, Greenwood Village, USA

**Keywords:** complicated parapneumonic effusion, c-reactive protein, crp, intrapleural fibrinolytic therapy, pleural empyema, video-assisted thoracoscopic surgical decortication

## Abstract

Treatment endpoints of empyema thoracis lack specificity when determining clinical success. While a declining C-reactive protein (CRP) may indicate a response to treatment, the expected rate of decline is not well-established. An observational analysis was performed on patients who were treated for either a parapneumonic effusion or empyema by tube thoracostomy and possessed at least two sequential CRP measurements. CRP trends were retrospectively modeled in a cohort of 104 patients. The rate of CRP decline was affected by the use of intrapleural enzymatic therapy, baseline CRP, and the value of the pleural pH. CRP declined at a daily rate of 12.3% in those who received intrapleural enzymatic therapy (IET) and by 17.8% in those who did not, but frequently remains elevated at discharge, thus lacking specificity as an isolated biomarker in determining treatment success or failure.

## Introduction

Patients who are diagnosed with a parapneumonic effusion or empyema carry an excellent prognosis with the use of intrapleural enzymatic therapy (IET). However, the disease process often necessitates prolonged hospitalization, invasive procedures, and serial imaging [1]. Current endpoints of treatment, such as the resolution of fever, resolution of leukocytosis, improvement in pleural opacity on imaging, and a low chest tube output, lack specificity in assessing a response to therapy. A reduction in C-reactive protein (CRP), an acute phase protein produced as a result of inflammation or tissue injury, has been used as a supplementary endpoint of treatment. Yet, the expected rate of decline and time to normalization have not been established, limiting its clinical utility.

CRP has been utilized in diagnosing the etiology of pleural effusions [2,3], to predict mortality [4], and to predict surgical success [5]. However, literature regarding the expected trend in CRP among patients treated with medical management is limited. A retrospective observational analysis was conducted to evaluate trends in CRP levels in patients undergoing treatment for parapneumonic effusion or empyema.

## Materials and methods

Data set

This was a retrospective descriptive study of patients with confirmed parapneumonic effusion or empyema who were treated by ultrasound-guided small-bore tube thoracostomy. Inclusion criteria included adults aged 18 years or older who were hospitalized between January 2015 and January 2025, with a primary diagnosis of parapneumonic effusion or empyema, who required tube thoracostomy, and who possessed both a CRP value at the time of tube insertion and at least one additional value prior to its removal. CRP was measured using quantitative immunoturbidimetry. An institutional review board waiver was obtained from the Intermountain Health Privacy Board (IRB number: 1053261).

All patients treated with IET received a regimen of alteplase 10 mg and dornase 5 mg concurrently instilled every 12 hours for a duration of one hour for three consecutive days. Those who did not receive IET were treated routinely with 30 mL of 0.9% normal saline flushes every 12 hours to maintain patency of the tube. The insertion and removal of the chest tube was at the discretion of the consulting pulmonologist. Surgical intervention was performed at the discretion of both the consulting pulmonologist and a thoracic surgeon.

Data collected

Age, gender, duration of tube thoracostomy, CRP, presence of bacteremia, pleural culture, pleural pH, pleural lactate dehydrogenase (LDH), pleural glucose, presence of empyema, white blood cell count (WBC), use of IET, death, and surgical intervention.

Outcomes and measures 

Study Groups

Patients who did not receive IET and patients who received IET within the first five days of their hospital stay. In the mixed-effects regression model, CRP values obtained on days preceding the initiation of IET were included with those who never received IET. All CRP values following the completion of IET were included in the IET-treated cohort. 

Primary Outcomes

The rate of serum CRP decline in the first nine days following chest tube insertion, and the time until a 50% reduction or value less than 50 mg/L.

Secondary Outcomes

Rates of death and surgical intervention.

Statistical analysis

Statistical analysis was performed using Stata software (version 16.1, StataCorp LLC, College Station, TX, US). Normality of continuous variables was assessed using the Shapiro-Wilk test. Missing data were handled by listwise deletion. Data collection ended at the occurrence of either death, discharge, or surgery. Baseline continuous variable mean differences were detected using an unpaired t-test, while ordinal variable differences were determined using Fisher’s exact test.

A linear mixed-effects regression model was used to account for repeated logarithmic CRP measurements within patients and adjusted for confounders. The model was fitted using maximum likelihood estimation. Huber-White robust standard errors were used, adjusting for heteroskedasticity and misspecification of the covariance structure. Covariates of the model included: age, gender, baseline CRP, baseline WBC, pleural pH, pleural LDH, pleural glucose, the presence of empyema by visual appearance, the presence of bacteremia, and bacterial growth within the pleural fluid culture. Day zero of CRP corresponded to the day of chest tube insertion. To assess whether the post-treatment CRP slopes differed, a joint Wald test of the treatment coefficients was performed. A p-value less than 0.05 was considered statistically significant.

## Results

Baseline demographics

A total of 104 patients were included in the analysis, including 67 who received IET and 37 who did not (Table 1).

**Table 1:** Baseline characteristics CRP: C-reactive protein; WBC: White blood cell count. All values are expressed as mean ± standard deviation. Continuous variable mean differences were determined using an unpaired t-test, and ordinal variable differences were determined using Fisher’s exact test.

	No IET (n=37)	IET (n=67)	p-value	Statistical method
Age (years)	60.27 ± 15.27	61.48 ± 16.91	0.72	Unpaired t-test
Male (N (%))	22 (59.46)	43 (64.18)	0.68	Fisher’s exact test
Pleural pH	7.43 ± 0.26	7.28 ± 0.25	0.01	Unpaired t-test
Pleural glucose (mg/dL)	73.32 ± 60.32	53.11 ± 54.92	0.10	Unpaired t-test
Pleural lactate dehydrogenase (U/L)	1289.73 ± 1330.62	1684.34 ± 1356.99	0.18	Unpaired t-test
Empyema (N (%))	8 (21.62)	12 (17.91)	0.80	Fisher’s exact test
Pleural fluid bacteria isolated (N (%))	16 (43.24)	33 (49.25)	0.68	Fisher’s exact test
Bacteremia (N (%))	4 (10.81)	13 (19.40)	0.41	Fisher’s exact test
Baseline CRP (mg/L)	178.89 ± 80.45	213.67 ± 85.39	0.05	Unpaired t-test
Baseline WBC (K/µL)	12.97 ± 6.75	18.51 ± 10.43	0.01	Unpaired t-test

The analysis included 749 observations, with an average of 7.2 observations per patient. 

Of those who received IET, 17 began on day zero, 18 on day one, 15 on day two, 12 on day three, and five on day four. The two groups differed only in baseline serum CRP, baseline serum WBC, and pleural pH. 

**Figure 1:**
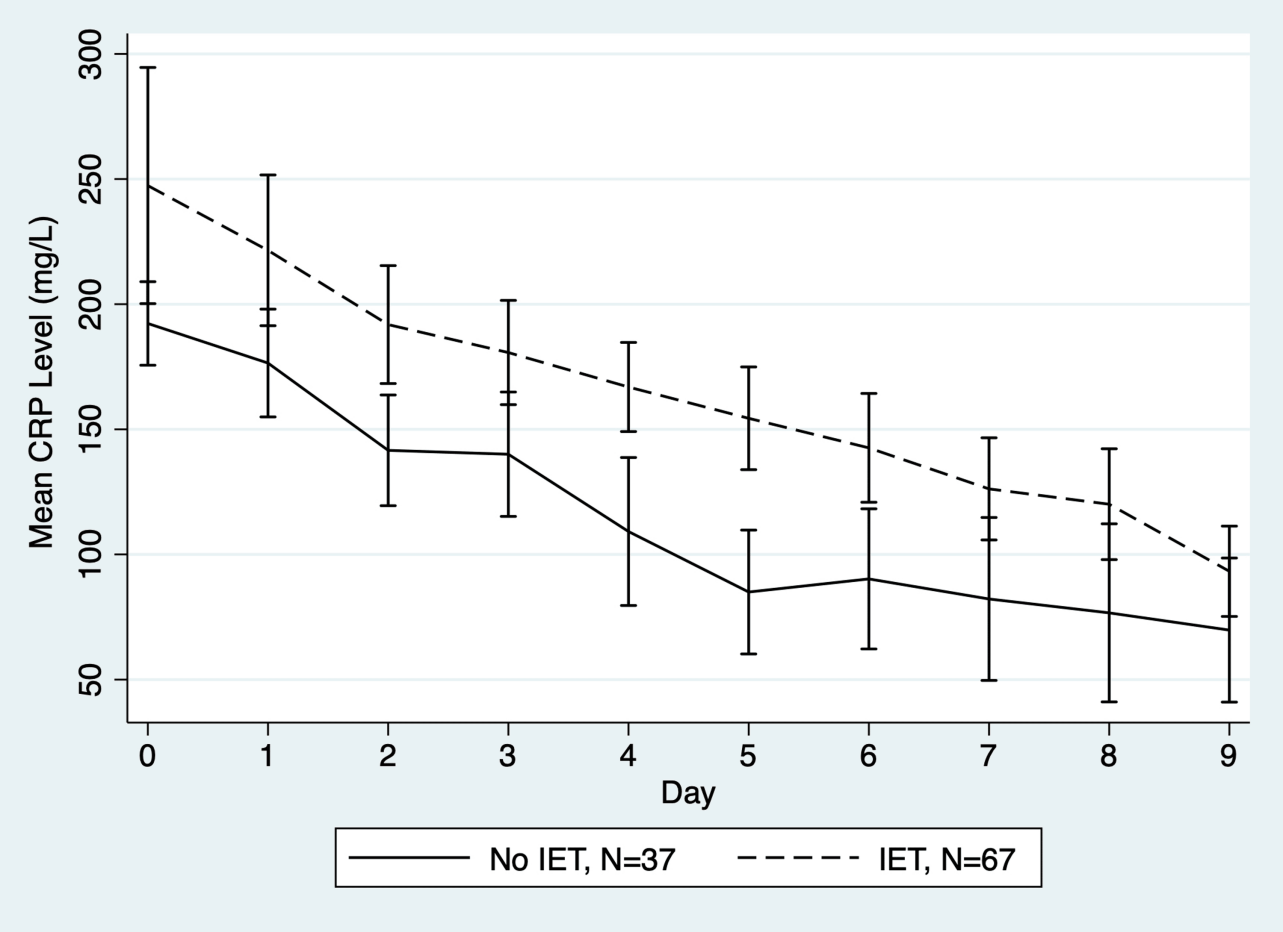
Mean daily CRP IET: intrapleural enzymatic therapy; CRP: C-reactive protein. Pre-intervention data are included in the “No IET” group. Image credit: Figure created by the authors using Stata software (version 16.1, StataCorp LLC, College Station, TX, US).

The median duration of chest tube drainage was six days (interquartile range 2-19). Baseline CRP was similar in those diagnosed with empyema (192.20 mg/L, 95% CI (147.67, 236.72)) to those diagnosed with a complicated parapneumonic effusion (203.48 mg/L, 95% CI (185.50, 221.44)), p=0.60. The trend of unadjusted CRP values with and without the use of IET are demonstrated in Figure 1.

Isolated pleural bacteria included those of the *Streptococcus anginosus *group (21 cases), anaerobes including *Fusobacterium spp., Prevotella spp., Bacteroides spp., Parvimonas spp.* (10), *Staphylococcus aureus* (6), *Streptococcus pneumoniae* (4), *Escherichia coli* (3), and other gram-negative (5) and gram-positive bacteria (6). 

Primary outcomes

CRP Decline

**Figure 2:**
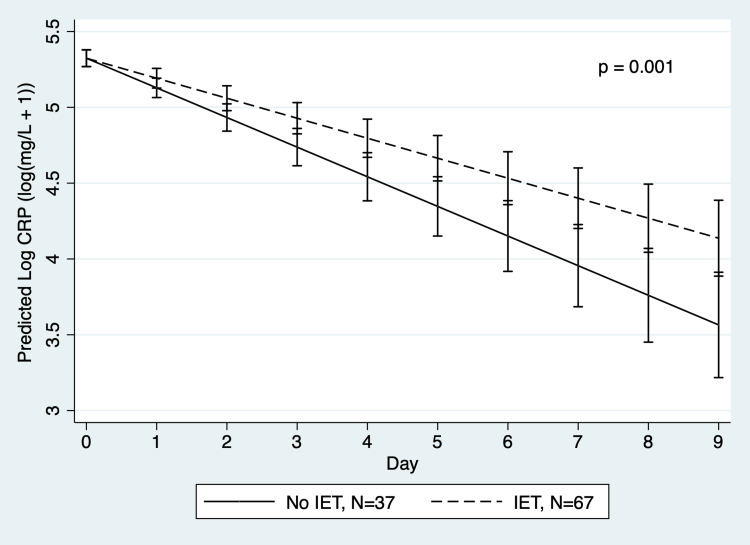
Mean serum CRP value by days from tube thoracostomy insertion in a mixed regression model IET: intrapleural enzymatic therapy; CRP: C-reactive protein. Pre-intervention data are included in the “No IET” group. Image credit: Figure created by the authors using Stata software (version 16.1, StataCorp LLC, College Station, TX, US).

A linear mixed-effects regression of log-transformed CRP over days zero through nine exhibited a mean daily decline of 17.8%, 95% CI (14.5%, 20.9%) in those who did not receive IET, and by 12.3% per day, 95% CI (9.0%, 15.6%) in those who received IET, p=0.001 (Figure 2). 

For each 1 mg/L increment in baseline serum CRP, there was a corresponding rise in CRP by 0.44%, 95% CI (0.37%, 0.51%). Similarly, an increase in baseline serum WBC by 1000 cells per microliter was associated with an elevation in CRP by 0.64%, 95% CI (0.01%, 1.27%). Each 0.1 unit decrease in pleural pH increased CRP by 3.22%, 95% CI (0.12%, 6.63%). Conversely, the presence of empyema was associated with a reduction in CRP by 20.68%, 95% CI (4.3%, 34.3%). No other individual covariate was significantly associated with CRP values in the model (Table 2).

**Table 2:** Covariates in the mixed linear regression model with peripheral CRP outcome per day IET: intrapleural enzymatic therapy; LDH: lactate dehydrogenase; CRP: C-reactive protein; WBC: white blood cell count.

Variable	Coefficient	Standard error	p-value	95% CI
Day	-0.1954	0.0198	0.000	-0.2343, -0.1566
IET * time	0.0636	0.0194	0.001	0.0254, 0.1017
Age	-0.0017	0.0019	0.38	-0.0054, 0.0021
Gender	-0.0196	0.0573	0.73	-0.1320, 0.0928
Day of IET initiation	-9.05 x 10^-5^	6.52 x 10^-5^	0.17	-2.183 x 10^-5^, 3.73 x 10^-5^
Pleural pH	-0.3263	0.1605	0.04	-0.6408, -0.0118
Pleural glucose	0.0001	0.0007	0.88	-0.0013, 0.0015
Pleural LDH	-4.63 x 10^-5^	2.9 x 10^-5^	0.11	-1.032 x 10^-5^, 1.06 x 10^-5^
Pleural bacteria	-0.0135	0.0701	0.85	-0.1509, 0.1240
Bacteremia	-0.0857	0.0719	0.23	-0.2265, 0.0552
Empyema	-0.2317	0.0957	0.02	-0.4192, -0.0442
Baseline CRP	0.0044	0.0004	0.000	0.0037, 0.0051
Baseline WBC	0.0064	0.0032	0.05	0.0001, 0.0127
Constant	6.9487	1.2434	0.000	4.5117, 9.3858

*CRP Values at Discharge* 

Among the 94 patients (90.38%) who survived to discharge without surgical intervention, the mean CRP on the day of discharge was 88.57 mg/L, 95% CI (76.87, 100.26). Of these patients, 67 (71.28%) achieved a reduction of CRP from baseline of at least 50%, and 24 patients (25.53%) achieved a CRP less than 50 mg/L at the time of discharge. No patients required readmission for surgery.

Secondary outcomes

Hospital Mortality

Three patients (2.88%) died, once on each of hospital days one, two, and four, occurring solely in those who received IET (p=0.55). The mean baseline CRP in non-survivors (176.67 mg/L, 95% CI (15.36, 337.97)) was similar to those who survived to discharge (202.03 mg/L, 95% CI (185.13, 218.93)), p=0.61. 

Surgical Intervention

Six patients (5.77%) underwent surgical intervention by video-assisted thoracoscopic surgery (VATS), all who received IET (p=0.09), with a median duration of nine days after tube thoracostomy. Baseline serum CRP was similar in those who required surgery (244.25 mg/L, 95% CI (198.23, 290.27)) to those who did not (198.67 mg/L, 95% CI (181.37, 215.96)), p=0.20. The mean CRP on the day of the surgery was 151.63 mg/L, 95% CI (67.00, 236.26). No patients with purulent empyema required surgery. 

## Discussion

CRP is a widely used biomarker in pleural infection, yet its utility in guiding treatment remains complex. While pleural fluid evacuation and antibiotic therapy typically reduce CRP values, despite effective drainage and clinical improvement, a persistent elevation of CRP is common at the time of hospital discharge. Persistent elevation of inflammatory markers may complicate clinical decision-making, potentially delaying hospital discharge or prompting surgical intervention. For instance, one study reported CRP mean values of 116 mg/L even following definitive surgical treatment at postoperative day seven [5]. In fact, the rate of decline of CRP was similar between those considered to have surgical success and those who did not [5]. This value exceeded that of our cohort at the time of discharge, and is only slightly higher than the mean CRP of our cohort on the day of surgery. 

In our model, IET was associated with a slower CRP decline, likely reflecting a selection bias toward more severe cases rather than treatment-induced inflammation. Notably, a prospective evaluation of 30 IET-treated patients demonstrated 90% efficacy, with a median CRP decreasing from 115 mg/L to 50 mg/L by day seven [6]. Similarly intrapleural alteplase without dornase reduced CRP from 126.4 mg/L to 31.5 mg/L in 20 consecutive patients [7]. Pleural fluid pH, an established predictor of outcomes in pleural infection, significantly influenced CRP values. Low pleural pH was associated with a markedly reduced rate of CRP decline, reflecting greater severity. Paradoxically, the presence of empyema led to a more rapid recovery, which is consistent with the notion that the presence of empyema may portend a better prognosis [8].

An upward trend in CRP has been associated with an increased mortality in one large model of empyema treatment [4], though this was not observed in our study due to low mortality rates. Likewise, among patients hospitalized with a primary diagnosis of pneumonia, a one-day increment in CRP greater than 50 mg/L was associated with a greater severity of illness or bacteremia [9], while survival has been linked to a 50% reduction in CRP [10]. Persistent CRP values greater than 100 mg/L on day four have also been associated with complications of pneumonia [11], while a failure to achieve a 50% reduction by day four is associated with an increased mortality [12]. However, while serum CRP values in parapneumonic infection exceed that of uncomplicated pneumonia, the rate of decline may be slower, limiting direct comparisons. The favorable prognosis of pleural space infection, driven by less virulent pathogens, further complicates the interpretation of CRP and its decline as a predictor of mortality.

In our cohort, no deaths or surgical intervention occurred among patients who did not receive IET. Of the three patients who did not survive to discharge, all were transitioned to hospice care and no deaths were an immediate consequence of pleural sepsis. Six patients underwent surgical intervention, out of which three were performed due to persistent pleural fluid on imaging, one for lung entrapment, and two for hemorrhagic complications related to IET. Surgical utilization overall was low, however, the decision to undergo surgical intervention is highly biased by numerous patient and provider factors. 

This retrospective analysis is limited by the variable IET initiation timing and treatment selection bias, as there are currently no standardized criteria regarding the use of IET. To mitigate these limitations, the mixed regression model was utilized to lessen bias and variance of practice preferences. Patients with lower pleural pH and higher baseline CRP values were more likely to receive IET, suggesting provider bias when determining the need for IET. The favorable prognosis of pleural space infection complicates the identification of covariates that predict success, particularly when evaluated with a modest sample size. Finally, the wide variance of CRP values underscores the variability of the disease process, driven by the extent of pleural fluid loculation, the duration of illness, and the causative pathogen.

## Conclusions

A declining CRP is an insufficient standalone indicator of treatment success in parapneumonic effusion and empyema. Despite over 90% of patients achieving hospital discharge without surgical intervention, CRP declined by 50% in only three-quarters of the cohort, while only one-quarter of patients achieved a reduction to less than 50 mg/L. While serial monitoring of CRP provides valuable supplementary data, its lack of specificity limits its utility as an isolated endpoint of treatment. Clinicians should integrate CRP trends with other clinical findings to optimize decision-making and endpoints of treatment.
